# Understanding the Microphysical Control and Spatial‐Temporal Variability of Warm Rain Probability Using CloudSat and MODIS Observations

**DOI:** 10.1029/2022GL098863

**Published:** 2022-05-24

**Authors:** Zhibo Zhang, Lazaros Oreopoulos, Matthew D. Lebsock, David B. Mechem, Justin Covert

**Affiliations:** ^1^ Physics Department UMBC Baltimore MD USA; ^2^ Goddard Earth Sciences Technology and Research II UMBC Baltimore MD USA; ^3^ Climate and Radiation Laboratory NASA Goddard Space Flight Center Greenbelt MD USA; ^4^ Jet Propulsion Laboratory California Institute of Technology Pasadena CA USA; ^5^ Department of Geography & Atmospheric Science University of Kansas Lawrence KS USA

**Keywords:** warm rain, probability of precipitation, CloudSat, MODIS

## Abstract

By combining measurements from MODIS and the CloudSat radar, we develop a parameterization scheme to quantify the combined microphysical controls by liquid water path (LWP) and cloud droplet number concentration (CDNC) of the probability of precipitation (PoP) in marine low cloud over tropical oceans. We demonstrate that the spatial‐temporal variation of grid‐mean in‐cloud <PoP> can be largely explained by the variation of the joint probability density function of LWP and CDNC in the phase space specified by the bivariate PoP (LWP and CDNC) function. Through a series of sensitivity tests guided by this understanding, we find that in the Southeastern Pacific and Atlantic the stratocumulus to cumulus transition of the <PoP> is mainly due to the variation of CDNC while the annual cycle is mainly due to the variation of LWP. The results of this study provide a viable way to diagnose the root cause of warm rain problems in global climate models.

## Key Points

- A parameterization scheme developed to quantify variation of warm rain probability as a function of cloud droplet number and water path
- The transition of warm rain probability from stratocumulus to cumulus regime is associated more strongly with variation of droplet number
- The annual cycle of warm rain probability is associated more strongly with cloud water path variations

## 1 Introduction

Marine low cloud (MLC) covers a significant fraction of the Earth's surface and plays a critical role in modulating the global radiative energy balance (Klein & Hartmann, 1993; Wood, 2012). The focus of this study is on warm rain processes in MLC that strongly influence the total water budget and therefore the lifetime and radiative effects of MLC (Albrecht, 1989; Kubar et al., 2009; Stevens et al., 1998). Observations from the CloudSat radar and other A‐Train satellite sensors such as MODIS (Moderate Resolution Spectroradiometer) provide rich information on cloud and precipitation properties on a global scale. Lebsock et al. (2008) and L’Ecuyer et al. (2009) studied the impacts of aerosols on MLC using the combination of CloudSat, MODIS, and other satellite observations. The concept of probability of precipitation (PoP) proposed in these studies was adopted in many later studies to quantify the susceptibility of warm rain to aerosols and to evaluate GCM simulations (e.g., Mann et al., 2014; Mülmenstädt et al., 2020; Song et al., 2018; Wang et al., 2012). A common conclusion from these studies is that the PoP tends to increase with liquid water path (LWP) and decrease with cloud droplet number concentration (CDNC), which together largely explains the covariation of PoP with aerosols. Here, we build upon the previous studies and investigate an important question: To what extent does the dependence of PoP on LWP and CDNC explain the spatiotemporal variations of PoP over the tropical oceans? More specifically, we are interested in the relative role of LWP and CDNC in regulating the spatial variation of PoP as one transitions from the coastal stratocumulus (Sc) region to remote ocean cumulus (Cu) region and the annual variation of PoP in these regions. For this purpose, we use collocated CloudSat and MODIS observations from 2009 to 2019 (see Section 2). We develop a parameterization scheme to quantify the dependence of PoP on LWP and CDNC (see Section 3) and investigate the spatiotemporal variation of PoP in three Sc‐Cu regions, namely the Southeastern Pacific (SEP), Northeastern Pacific (NEP), and Southeastern Atlantic (SEA) (see Section 4). The results and implications are discussed in Section 5.

## 2 Data and Methodology

Following the previous studies, we define the grid‐mean in‐cloud <PoP> for a 1° latitude × 2° longitude grid box as the ratio of the precipitating columns *N*_precip_ to the total number of MLC columns *N*_MLC_ within the grid over the period of interest (e.g., month or year), that is, 〈PoP〉 = *N*_precip_/*N*_MLC_ (L’Ecuyer et al., 2009; Mann et al., 2014; Wang et al., 2012). We use the combination of CloudSat Cloud Precipitation Radar (CPR), CALIPSO Cloud‐Aerosol Lidar with Orthogonal Polarization, and MODIS observations to identify MLC because of CPR alone missing a large fraction of MLC due to radar surface clutter (Christensen et al., 2013) and the fact that many MLC fall below its detection threshold (Rapp et al., 2013). Specifically, we first use the 2B‐GEOPROF‐LIDAR product derived from the combined CloudSat and CALIPSO observations (Mace & Zhang, 2014) to identify low clouds with cloud top height lower than 3 km. To ensure that cloud phase is liquid, we use the collocated MODIS retrievals (from the MYD06 product) in CloudSat's MODIS‐AUX product to select CloudSat columns with cloud top phase labeled as liquid and cloud top temperature warmer than 273 K. The precipitating MLC columns *N*_precip_ are identified using CloudSat's 2B‐GEOPROF product. An MLC column is considered to be precipitating if the maximum radar reflectivity (dBZ_max_) ￼ −15. This simple threshold‐based classification has been used in several previous studies (e.g., L’Ecuyer et al., 2009; Mann et al., 2014; Wang et al., 2012); ￼ dBZ_max_ > −15 is also a key criterion for identifying drizzling cloud over the ocean in the CloudSat operational retrieval (Haynes et al., 2009). The use of dBZ_max_ can also facilitate the comparison between CloudSat observations and GCM simulations through satellite simulators (Kay et al., 2018; Song et al., 2018).

**Figure 1:**
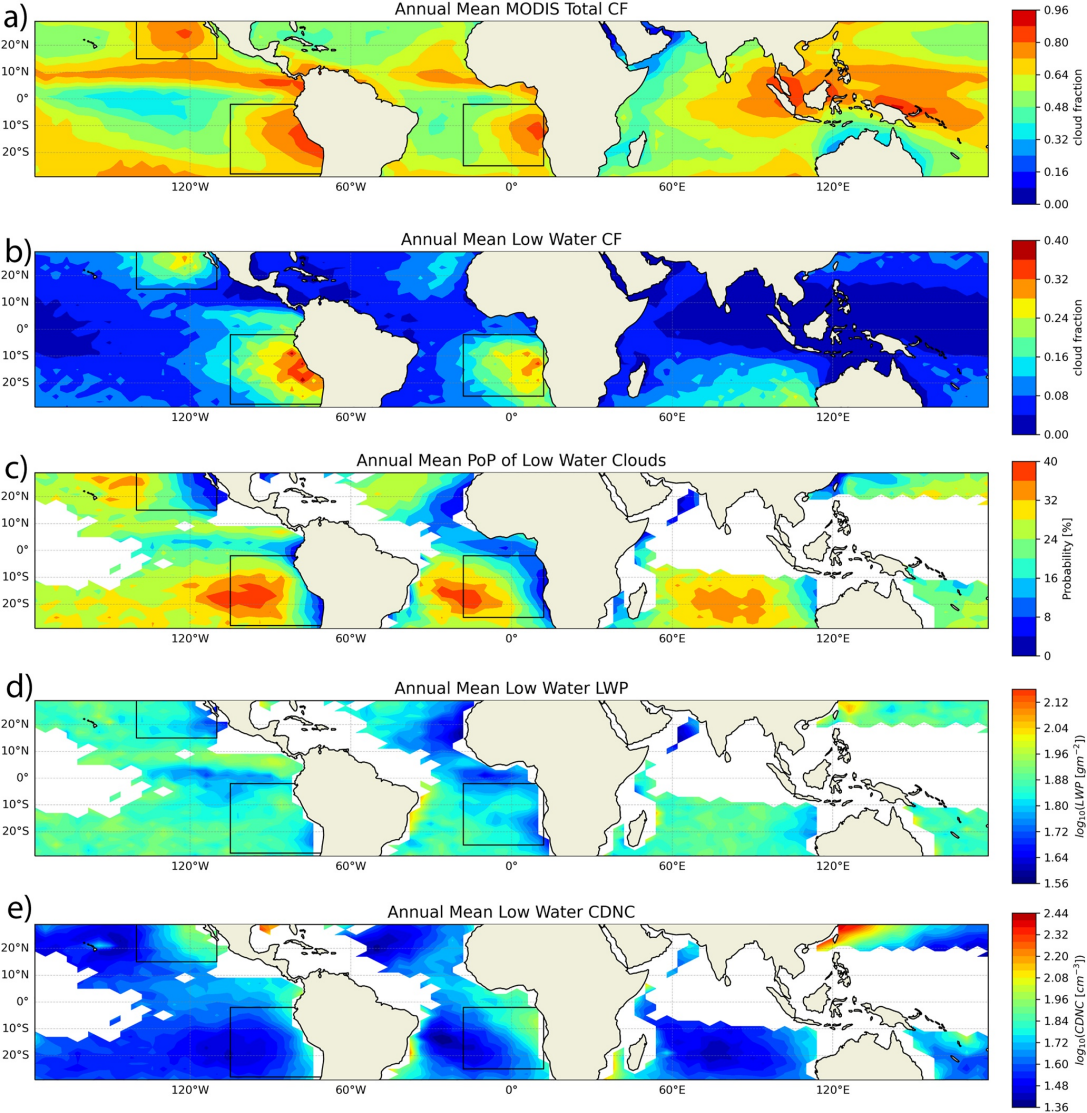
Annual mean (a) total and (b) Marine low cloud (MLC) cloud fraction, (c) probability of precipitation, (d) liquid water path, and (e) cloud droplet number concentration of MLC derived from CloudSat and MODIS observations. Regions with MLC fraction below 5% are masked in white in panels (c, d, and e) for quality assurance.

Figures 1a and 1b show the total and MLC cloud fraction (CF) over the tropical ocean derived from these products. As expected, a high MLC fraction is found mainly in the eastern parts of the major ocean basins, where the relative cold sea surface temperature and high lower tropospheric stability favor semipermanent Sc‐Cu desks extending thousands of kilometers (Klein & Hartmann, 1993; Wood, 2012).

We select three regions, SEP, NEP, and SEA, marked by the black rectangles in Figure 1 for further study. Figure 1c shows the annual mean <PoP> over the tropical ocean. Focusing on the three selected regions, we observe a consistent transition behavior of <PoP>, increasing from ∼5 to 10% in the coastal Sc region to ∼30% in the open ocean Cu regions (see also Figure 4). Understanding this transition of <PoP>, particularly the relative role of LWP and CDNC, is one of the motivations for this study. For this purpose, we use the operational MODIS product to estimate the LWP (Figure 1d) and CDNC (Figure 1e) for MLC. Several studies have suggested that adiabatic cloud structure be assumed in the estimation of LWP from MODIS observations, which would differ from the operational MODIS LWP based on vertically homogenous cloud structure by a factor of 5/6 (Seethala & Horváth, 2010; Wood & Hartmann, 2006). This difference is small and has negligible impacts on our analysis. Details on the latest MODIS cloud retrieval algorithm can be found in Platnick et al. (2017) and the method for the CDNC retrieval has been described and validated in many previous studies (e.g., Bennartz, 2007; Grosvenor et al., 2018; Zhang et al., 2019). Cloud droplet number concentration shows a clear and consistent transition pattern, decreasing from coastal Sc to open ocean Cu region (see also Figure 3). This behavior of MLC microphysics, which could be combined effects of aerosol‐cloud interactions from the coastal region to the remote ocean, has been noted in several previous studies (e.g., Bennartz & Rausch, 2017; Grosvenor et al., 2018; Wood, 2012). In contrast, the variation of LWP is not so prominent and only after meridional averaging, a weak increase of LWP from Sc to Cu regions can be seen in Figure 4.

## 3 Microphysical Control of <PoP> by LWP and CDNC

Many previous studies have noted that <PoP> increases with LWP and decreases with CDNC. In this study, we attempt to achieve a more comprehensive and quantitative understanding of their combined effect and relative importance in regulating the spatiotemporal variation of <PoP>. Our study is based on the key hypothesis that the behavior of <PoP> can be explained by the combination of the microphysical control PoP(LWP and CDNC) and the joint probability density function (PDF) of LWP and CDNC—PDF (LWP and CDNC). The PoP(LWP and CDNC) is mainly controlled by microphysical processes, that is, collision‐coalescence, and is therefore relatively invariant in space and time. This dependence of PoP on LWP and CDNC is similar in commonly employed precipitation‐rate scaling relationships derived from observations (Comstock et al., 2004; Pawloska & Brenguier, 2003; VanZanten et al., 2005). The spatial‐temporal variation of <PoP> is mainly represented by the shape of the PDF(LWP and CDNC) over each region. Based on this hypothesis, we can write the <PoP> in each grid box in Figure 1c in an integral form as follows

(1) 〈PoP〉=∬PoP(LWP,CDNC)PDF(LWP,CDNC)dLWPdCDNC

where PoP(LWP and CDNC) is a universal function across all grid boxes.

**Figure 2:**
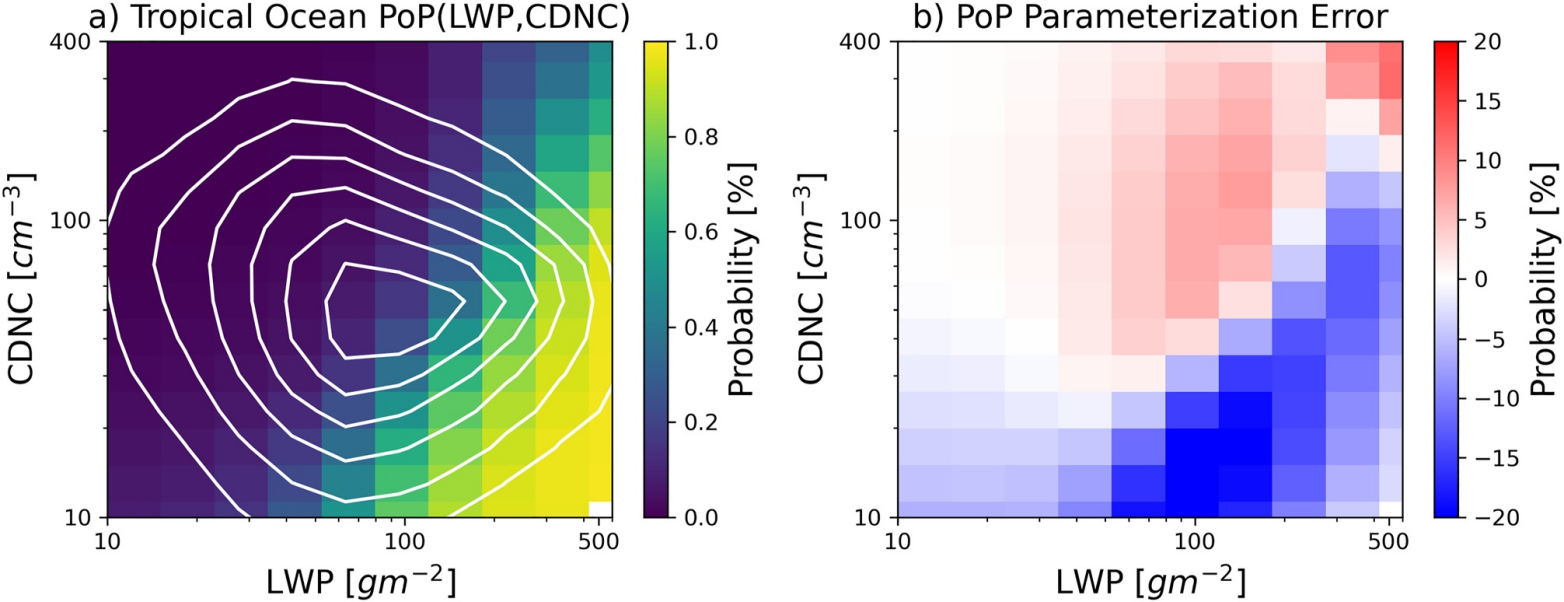
(a) The color scale indicates the probability of precipitation (PoP)(liquid water path (LWP) and cloud droplet number concentration (CDNC)) and the white contour lines show the normalized probability density function(LWP and CDNC) (maximum value normalized to unity) from 90% at the center to 15% at the edge with an interval of 15%. (b) Error of the parameterized PoP(LWP and CDNC) based on Equation 2 with respect to panel (a).

To test this hypothesis, we first derived the PoP(LWP and CDNC) based on the whole population of MLC over tropical oceans. The results are shown in Figure 2a. As expected, the PoP is close to zero where LWP is small and CDNC is large and approaches unity where LWP is large and CDNC is small. Similar trends have also been noted in previous studies (Kubar et al., 2009; Wang et al., 2012). However, these are rather infrequent conditions as the PDF(LWP and CDNC) (white contour lines in Figure 2a) indicates that MLC is most likely to have a moderate LWP around 90 gm^−2^ and CDNC around 50 cm^−3^. The orientation of the joint PDF also suggests that the LWP and CDNC of MLC over tropical oceans are not correlated. To further quantify the influences of LWP and CDNC on PoP, we fit the PoP(LWP and CDNC) in Figure 2a using the following bivariate logistic function

(2) $$\text{PoP}(x,y)=\frac{1}{1+\text{exp}\left[-\left(c_{0}+c_{1}x+c_{2}y\right)\right]}$$

where $x=\log_{10}\left(\frac{\text{LWP}}{1{\text{gcm}}^{-2}}\right)$ and $y=\log_{10}\left(\frac{\text{CDNC}}{1{\text{cm}}^{-3}}\right)$, respectively. The corresponding fitting coefficients are *c*_0_ = −6.9, *c*_1_ = 5.7, and *c*_2_ = −3.2. It should be noted that Equation 2 represents only a numerical regression not meant to capture underlying physics. Nevertheless, the function can approximate the PoP based on data reasonably well, with the error mostly within ±10% as shown in Figure 2b.

**Figure 3:**
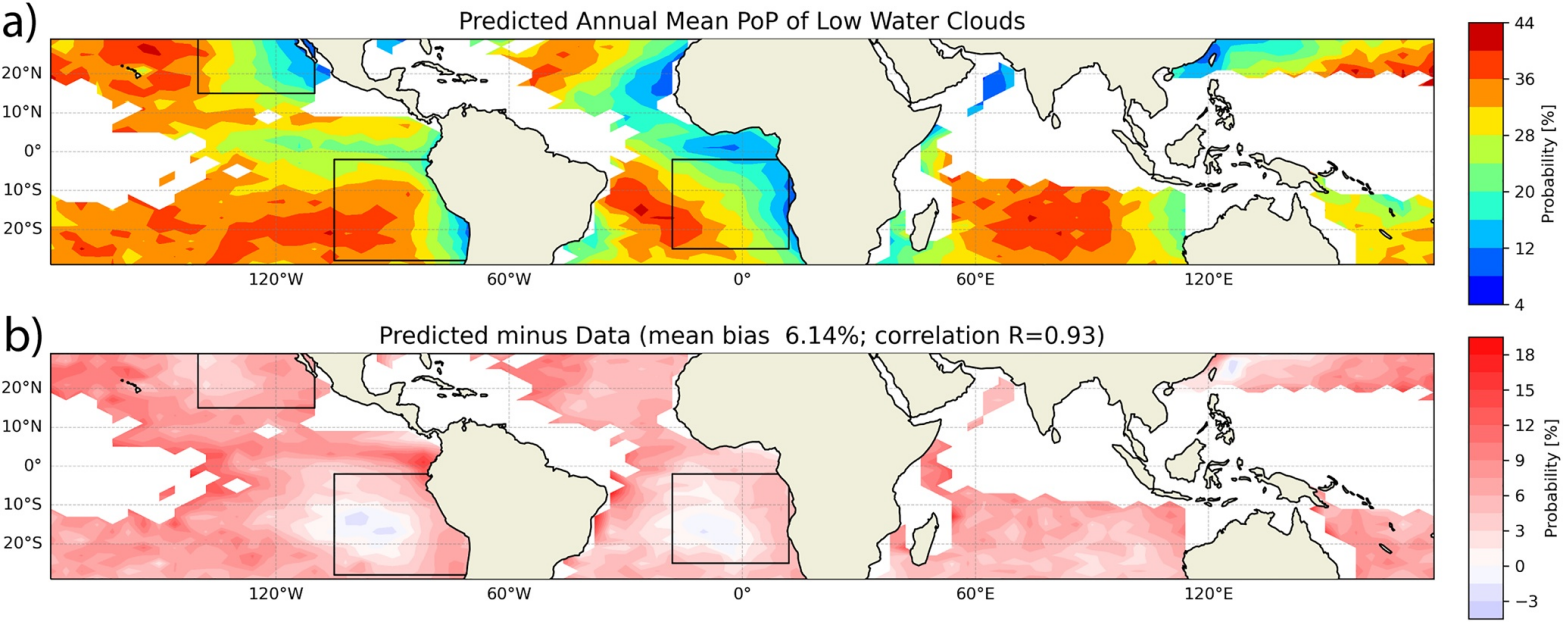
(a) <PoP> derived from Equation 1 based on the parameterized universal probability density function(liquid water path (LWP) and cloud droplet number concentration (CDNC)) and spatially varying probability density function (LWP and CDNC) of each grid. The error of the predicted <PoP> in panel (a) relative to the actual <PoP> is shown in Figure 1c.

With the PoP parameterization in hand, we can now test our hypothesis. First, we derive the annual mean PDF(LWP and CDNC) for each grid box based on the MODIS data. Then we insert the PDF(LWP and CDNC) of a given grid and the PoP(LWP andCDNC) parameterization of Equation 2 into Equation 1 to obtain the PoP for the grid. The resulting PoP is shown in Figure 3a and the error with respect to the <PoP> in Figure 1c is given in Figure 3b. Although the parameterization yields overestimates in most regions of low <PoP> and slight underestimates in regions of high <PoP>, the overall result is very encouraging. The predicted PoP captures the essential features of the data, especially the increasing trend of <PoP> from the coastal Sc region to the remote ocean Cu region. The reasonable agreement between Figure 3a and Figure 1c supports our hypothesis that PoP(LWP and CDNC) is mainly controlled by microphysical processes and therefore relatively invariant in space and time.

## 4 Spatial‐Temporal Variations of <PoP> in Selected Sc‐Cu Regions

**Figure 4:**
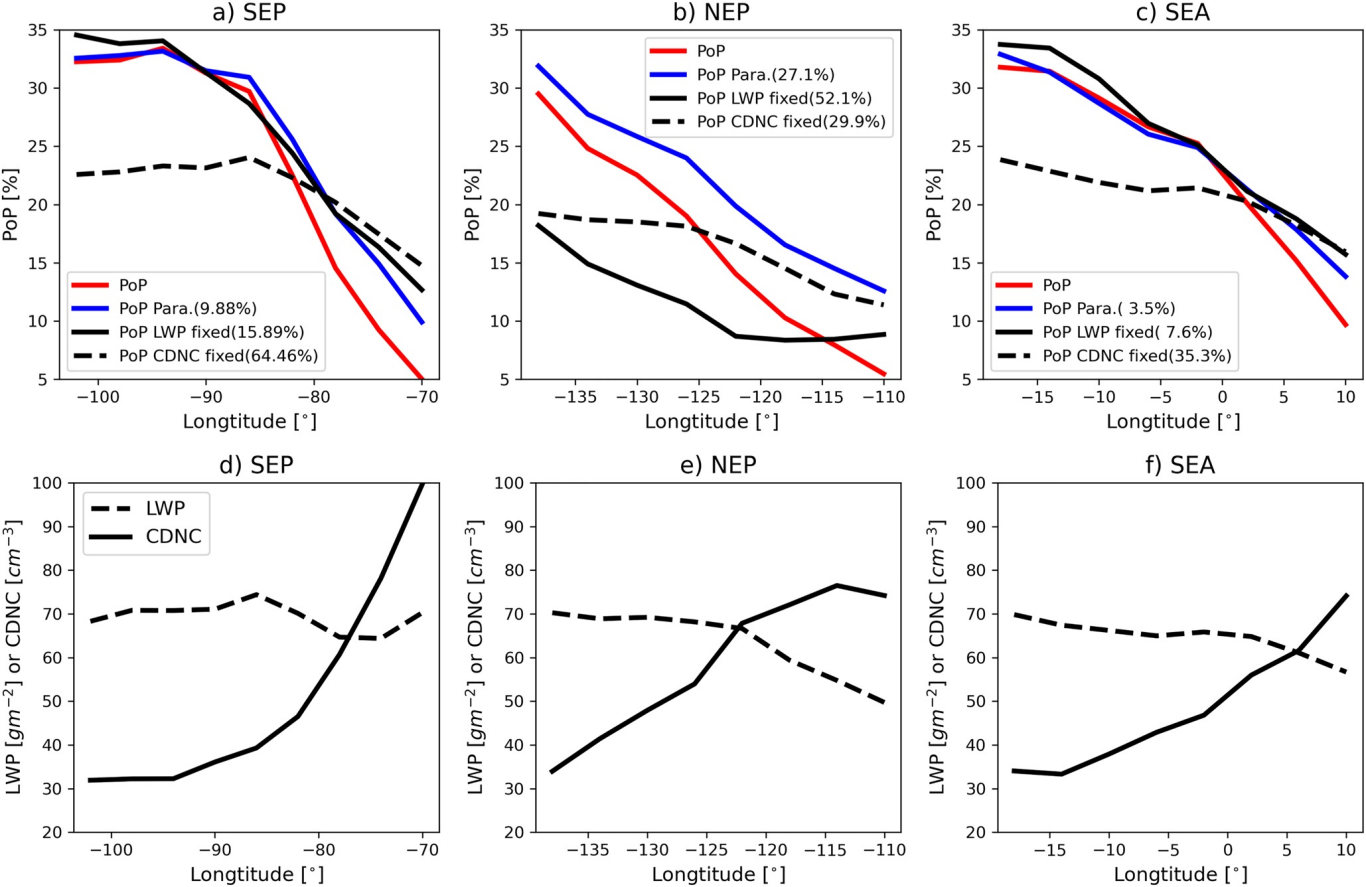
Meridional mean of <PoP> in the (a) Southeastern Pacific (SEP), (b) Northeastern Pacific (NEP), and (c) Southeastern Atlantic (SEA) regions as a function of longitude. Numbers in parenthesis are the root‐mean‐square error of different fitting results (see text for details). The meridional mean of liquid water path and cloud droplet number concentration in the (d) SEP, (e) NEP, and (f) SEA regions.

We now examine whether our understanding of the microphysical control of PoP can help us explain the spatiotemporal variations of <PoP> in the selected Sc‐Cu regions. First, we focus on the transition pattern of <PoP> from the coastal Sc region to the remote ocean Cu region. This transition pattern is clearly illustrated by the variation of the meridional mean of <PoP> with longitude in Figure 4. In all three regions, the meridional mean <PoP> increases from ∼5% to 10% to around 30%. In Figures 4d–4f, we plot the meridional mean LWP and CDNC for the three regions to understand their relative role in regulating the Sc to Cu transition of <PoP>. In all three regions, we observe a clear and significant decreasing trend of CDNC westward from coastal Sc region to remote ocean Cu region. This trend is especially prominent in the SEP region where the CDNC reduces by a factor of 3 from ∼100 cm^−3^ around 70°W to ∼30 cm^−3^ around 100°W. The decrease of CDNC is probably due to the combined effect of decreasing aerosol from the continental outflow region to the remote ocean and an increasing precipitation (coalescence) scavenging effect (Wood et al., 2012). In contrast, the LWP shows a general but moderate increase from the Sc to the Cu region. Note that all the LWP values in this study are averaged only over the cloudy portion (i.e., “in‐cloud”) of the grid box. Therefore, the increase of in‐cloud LWP seems to indicate an increase of cloud thickness and therefore of cloud water in individual cloud objects from Sc to Cu region even though the CF decreases.

Both the decrease of CDNC and increase of LWP favor the increase of <PoP>. But what is the relative importance of the two factors? To answer this question, we performed the following sensitivity study. First, as a sanity check we derived the longitudinal change of <PoP> in three regions based on the universal PoP(LWP and CDNC) parameterization and the meridional mean PDF(LWP and CDNC). The results are shown as blue curves in Figure 4 and are in good agreement with the <PoP> from observations (red lines). This further confirms our hypothesis that <PoP> can be understood as the combination of universal microphysical control and the geographical (latitudinal) variation of PDF(LWP and CDNC). Next, to investigate the relative role of LWP and CDNC, we derive two sets of <PoP> from Equation 1 based on the combinations of the universal PoP(LWP and CDND) and two different PDFs, one based on the marginal PDF(<LWP> and CDNC) and another based on the marginal PDF(LWP and <CDNC>), where <LWP> and <CDNC> correspond to the regionally averaged LWP and CDNC, respectively. The basic idea behind these tests is to gauge the relative importance of each variable. In both the SEP (Figure 4a) and SEA (Figure 4c), the <PoP> based on the PDF(<LWP> and CDNC) (solid black lines; referred to as “LWP fixed”) is in reasonable agreement with the <PoP> considering co‐variation of LWP and CDNC (blue lines), as well as the results based on observations. In contrast, as evidenced by the RMS errors the <PoP> based on the PDF(LWP and <CDNC>) (solid black lines; referred to as “CDNC fixed”) yields substantial underestimates relative to observations in the Cu region. Based on these results, we can conclude that in both regions, it is the decreasing trend of CDNC away from the coast that plays a more important role than the increasing trend of LWP in causing the transition pattern of <PoP>. The result in the NEP region (Figure 4b) is less clear. Both LWP fixed and CDNC fixed <PoP> show an increasing trend from the Sc region to Cu region but neither of them provides a sufficient explanation of <PoP>. This result indicates that the covariation of both variables must be considered when explaining the transition of <PoP> in the NEP region.

**Figure 5:**
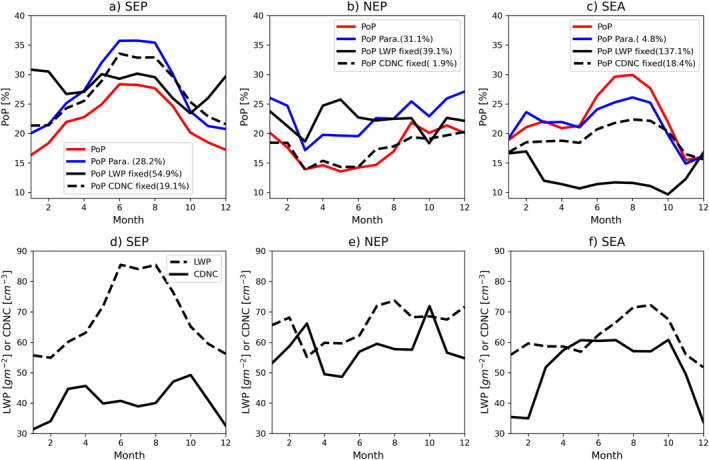
The annual cycle of regional mean <PoP> in (a) Southeastern Pacific (SEP), (b) Northeastern Pacific (NEP), and (c) Southeastern Atlantic (SEA). Numbers in parenthesis are the root‐mean‐square error of different fitting results (see text for details). The corresponding annual cycle of regional mean liquid water path and cloud droplet number concentration in (d) SEP, (e) NEP, and (f) SEA.

We now turn our attention to the annual cycle of <PoP>, which is shown in Figures 5a–5c for the three selected regions. In the SEP, the monthly mean <PoP> has a maximum around 28% in the austral winter and a minimum around 16% in the summer. The SEA region has a similar annual cycle. In both regions, the magnitude (maximum minus minimum) of the seasonal cycle is around 12%–15%. The NEP region has a significantly weaker annual cycle with a magnitude of only around 7%–8%. The regional means LWP and CDNC for the three regions are plotted in Figures 5d–5f. In both the SEP and SEA regions, LWP shows an annual cycle similar to that of the corresponding <PoP>, suggesting a dominant role of LWP in regulating the annual cycle of <PoP>. The CDNC in the SEP region exhibits two peaks, one in April and the other in October. In SEA, the CDNC remains relatively stable around 60 cm^−3^ from April to October and drops to around 35 cm^−3^ in December–February. In the NEP region, neither LWP nor CDNC shows a clear and significant annual cycle, which is not surprising given the weak <PoP> annual cycle in Figure 5b.

To quantify the relative role of LWP and CDNC variation in regulating the <PoP> annual cycle, we performed the same sensitivity test as the one used in understanding the Sc to Cu transition. As expected, the <PoP> predicted by holding CDNC constant at its annual mean value and allowing LWP to vary (i.e., “LWP only” dashed black lines in Figures 5a and 5c) captures the annual cycle of <PoP> in the SEP and SEA region very well, with a skill similar to that when allowing both to covary (blue lines) as evidenced by the RMS errors. In both regions, the <PoP> predicted by “CDNC only” (solid black lines) shows an annual cycle opposite to that of CDNC, as expected. In the SEP, the <PoP> predicted by “CDNC only” shows a peak in the austral winter months (July–August), which is in phase with that of <PoP> based on data, but agreement in other months is rather poor. In the SEA region, the <PoP> predicted by “CDNC only” is generally opposite to that of <PoP> based on observations. Based on these results, we conclude that the annual cycle of <PoP> in both the SEP and SEA is largely dominated by the variation of LWP. In the NEP region, the picture is less clear. While the <PoP> predicted by “LWP only” can help explain the minimum of <PoP> in the boreal spring to summer months (April–July), the <PoP> predicted by “CDNC only” provides a better explanation in the boreal fall and winter months (August–December). Therefore, there appears to be no dominant factor, and both LWP and CDNC need to be considered to explain the annual cycle of <PoP>.

Finally, it is important to note here that the above analysis only provides information about the correlation between <PoP> and cloud properties, it does not reveal a causal relationship. For example, the correlation between CDNC and <PoP> in Figure 4 could be interpreted as the influences of CDNC on PoP or the depletion of CDNC by precipitation. The underlying physics is beyond the scope of this study and left for future research.

## 5 Summary and Discussion

This study uses the combined power of CloudSat and MODIS observations to understand the microphysical control and spatiotemporal variations of the PoP (POP) of MLC in the tropics. First, we develop a bivariate nonlinear parameterization (Equation 2) to quantify the combined microphysical control of PoP by LWP and CDNC. We demonstrate that the spatiotemporal variation of <PoP> can be largely explained by the variation of the joint PDF of LWP and CDNC in the PoP(LWP and CDNC) phase space through the integral in Equation 1. Based on this understanding, we investigate the relative role of LWP and CDNC in regulating the Sc to Cu transition and the annual cycle of <PoP> in the SEP, NEP, and SEA regions. Through the “LWP only” versus “CDNC only” sensitivity tests, we find that in the SEP and SEA regions the increase of <PoP> from the Sc to Cu region is mainly due to the decrease of CDNC, while the annual cycle of the domain‐average <PoP> is controlled mainly by the variation of LWP. In the NEP region, there is not a dominant factor and both variables need to be considered in explaining the spatial‐temporal and seasonal variation of <PoP>.

The results in this study can be highly useful for the evaluation of MLC in GCMs. Many studies have shown that the PoP is too high in models, which could be an important reason for the long‐lasting MLC deficiency problem in GCMs (Mülmenstädt et al., 2020; Song et al., 2018). Our study provides a viable path to diagnose the root of the PoP problem. For example, by using satellite simulators, modelers can derive the PoP(LWP and CNDC) and the PDF(LWP andCDNC) based on model simulations, then compare the results to the observations shown in this study. The differences can help the modelers understand whether deficiencies in simulated <PoP> are mainly due to the microphysical control coming in microphysical schemes and subgrid parameterizations or the LWP and CDNC of MLC regulated by the model's large‐scale circulation and aerosol schemes.

## Data Availability

All the satellite data products used in this study are publicly available. CloudSat products are available from https://www.cloudsat.cira.colostate.edu/. MODIS cloud retrieval products are available from https://ladsweb.modaps.eosdis.nasa.gov/. Our Python code for deriving the probability of precipitation for marine low clouds from CloudSat and MODIS products is made publicly available through Zenodo (https://zenodo.org/badge/latestdoi/475052443).
